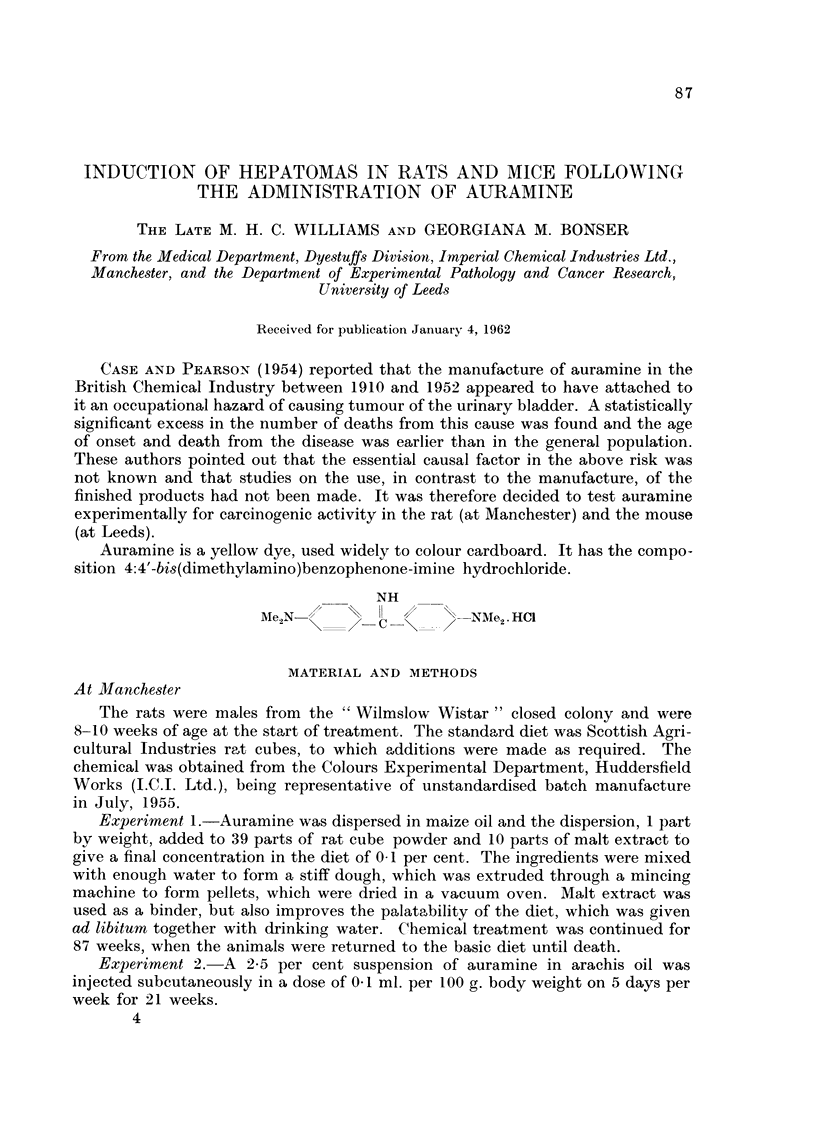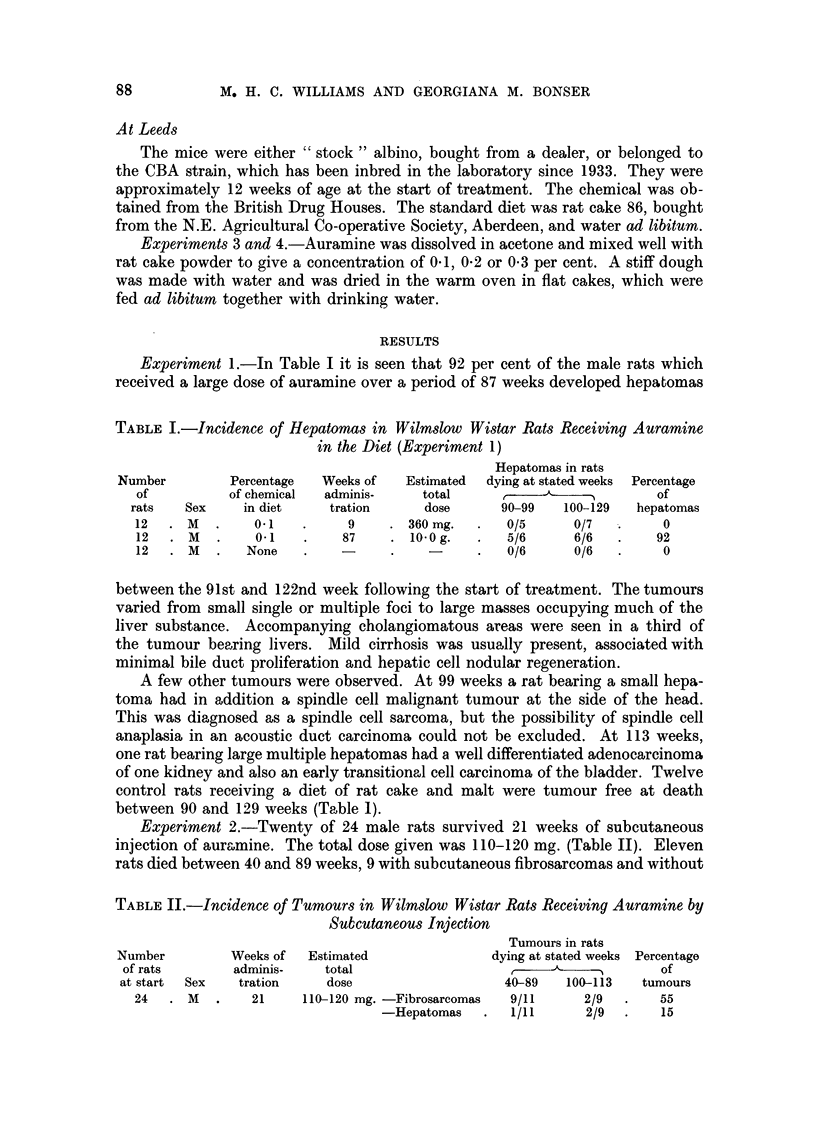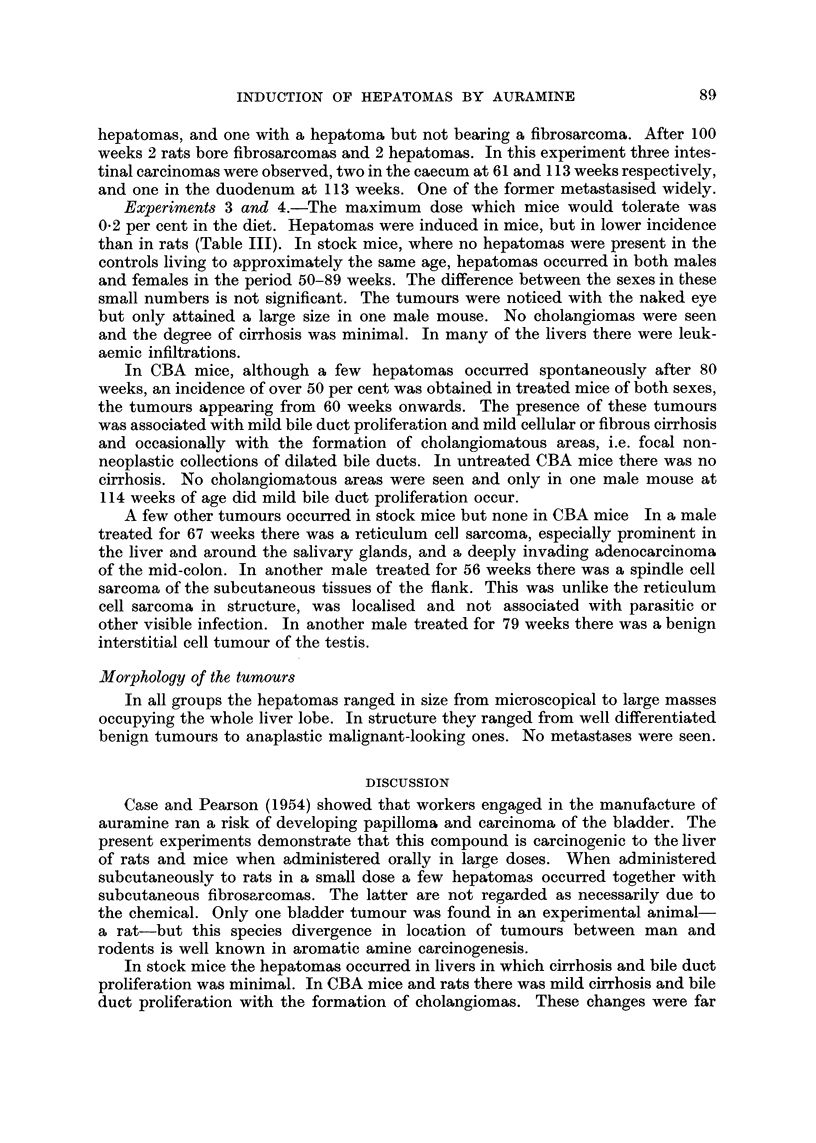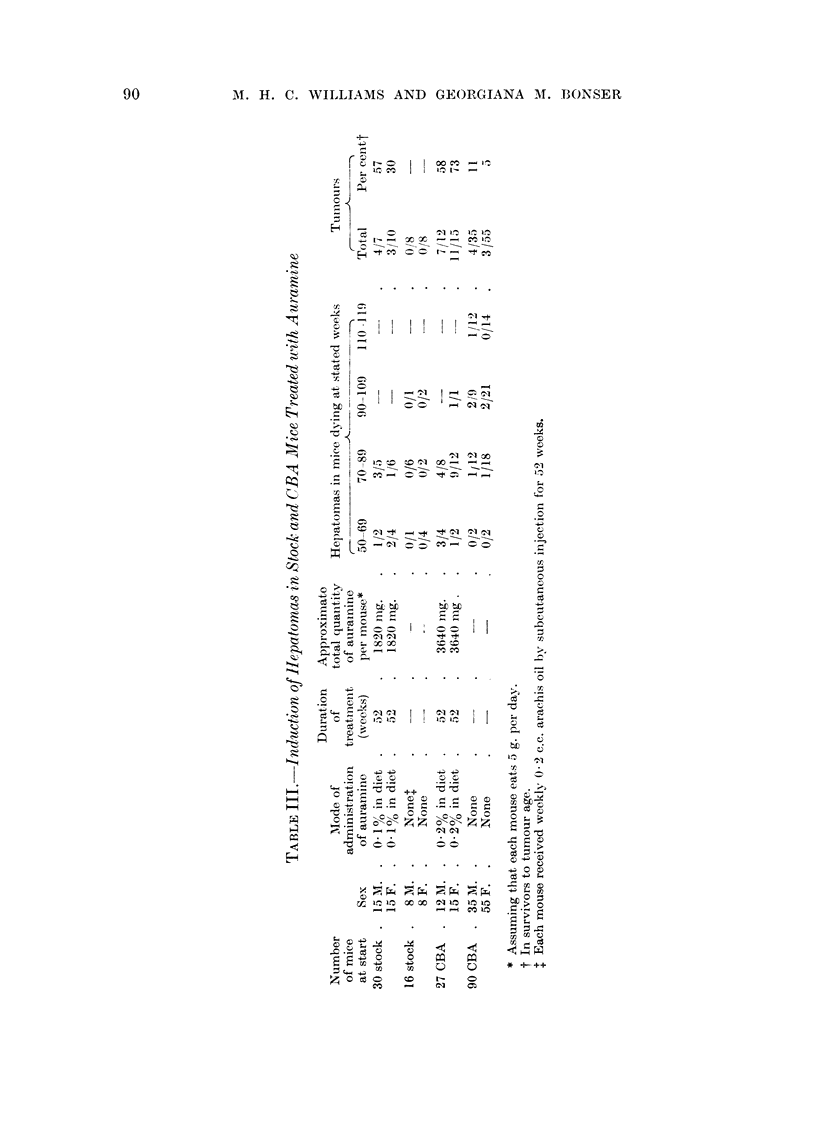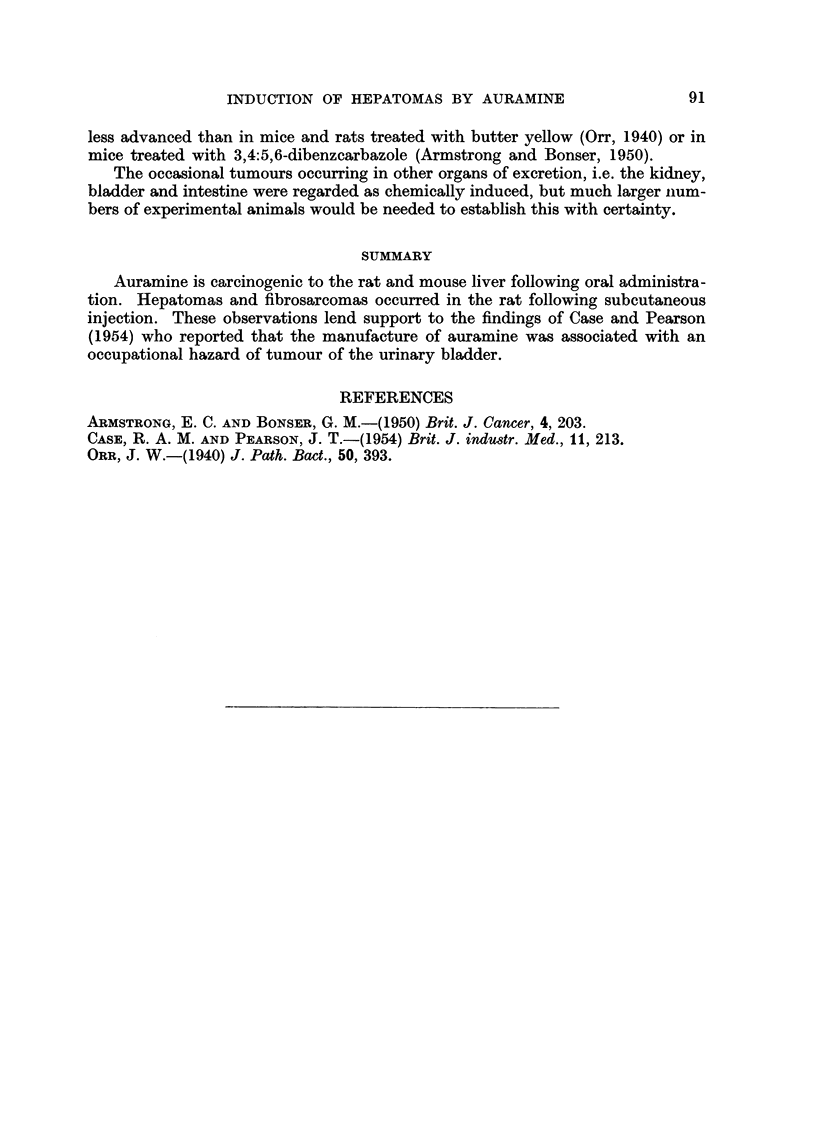# Induction of Hepatomas in Rats and Mice following the Administration of Auramine

**DOI:** 10.1038/bjc.1962.8

**Published:** 1962-03

**Authors:** M. H. C. Williams, Georgiana M. Bonser


### INDUCTION OF HEPATOMAS IN RATS AND MICE FOLLOWING THE ADMINISTRATION OF AURAMINE

THE LATE M. H. C. WILLIAMS AND GEORGIANA M. BONSER

*From the Medical Department, Dyestuffs Division, Imperial Chemical Industries Ltd., Manchester, and the Department of Experimental Pathology and Cancer Research, University of Leeds*

Received for publication January 4, 1962

CASE AND PEARSON (1954) reported that the manufacture of auramine in the British Chemical Industry between 1910 and 1952 appeared to have attached to it an occupational hazard of causing tumour of the urinary bladder. A statistically significant excess in the number of deaths from this cause was found and the age of onset and death from the disease was earlier than in the general population. These authors pointed out that the essential causal factor in the above risk was not known and that studies on the use, in contrast to the manufacture, of the finished products had not been made. It was therefore decided to test auramine experimentally for carcinogenic activity in the rat (at Manchester) and the mouse (at Leeds).

Auramine is a yellow dye, used widely to colour cardboard. It has the composition 4:4′-*bis*(dimethylamino)benzophenone-imine hydrochloride.

### MATERIAL AND METHODS

### *At Manchester*

The rats were males from the " Wilmslow Wistar " closed colony and were 8–10 weeks of age at the start of treatment. The standard diet was Scottish Agricultural Industries rat cubes, to which additions were made as required. The chemical was obtained from the Colours Experimental Department, Huddersfield Works (I.C.I. Ltd.), being representative of unstandardised batch manufacture in July, 1955.

*Experiment* 1.—Auramine was dispersed in maize oil and the dispersion, 1 part by weight, added to 39 parts of rat cube powder and 10 parts of malt extract to give a final concentration in the diet of 0·1 per cent. The ingredients were mixed with enough water to form a stiff dough, which was extruded through a mincing machine to form pellets, which were dried in a vacuum oven. Malt extract was used as a binder, but also improves the palatability of the diet, which was given *ad libitum* together with drinking water. Chemical treatment was continued for 87 weeks, when the animals were returned to the basic diet until death.

*Experiment* 2.—A 2·5 per cent suspension of auramine in arachis oil was injected subcutaneously in a dose of 0·1 ml. per 100 g. body weight on 5 days per week for 21 weeks.

*At Leeds*

The mice were either " stock " albino, bought from a dealer, or belonged to the CBA strain, which has been inbred in the laboratory since 1933. They were approximately 12 weeks of age at the start of treatment. The chemical was obtained from the British Drug Houses. The standard diet was rat cake 86, bought from the N.E. Agricultural Co-operative Society, Aberdeen, and water *ad libitum.*

*Experiments* 3 *and* 4.—Auramine was dissolved in acetone and mixed well with rat cake powder to give a concentration of 0·1, 0·2 or 0·3 per cent. A stiff dough was made with water and was dried in the warm oven in flat cakes, which were fed *ad libitum* together with drinking water.

### RESULTS

*Experiment* 1.—In Table I it is seen that 92 per cent of the male rats which received a large dose of auramine over a period of 87 weeks developed hepatomas

TABLE I.—*Incidence of Hepatomas in Wilmslow Wistar Rats Receiving Auramine in the Diet* (*Experiment* 1)

| Number of rats | Sex | Percentage of chemical in diet | Weeks of adminis-tration | Estimated total dose | Hepatomas in rats dying at stated weeks: 90–99 | 100–129 | Percentage of hepatomas |
|---|---|---|---|---|---|---|---|
| 12 | M | 0·1 | 9 | 360 mg. | 0/5 | 0/7 | 0 |
| 12 | M | 0·1 | 87 | 10·0 g. | 5/6 | 6/6 | 92 |
| 12 | M | None | — | — | 0/6 | 0/6 | 0 |

between the 91st and 122nd week following the start of treatment. The tumours varied from small single or multiple foci to large masses occupying much of the liver substance. Accompanying cholangiomatous areas were seen in a third of the tumour bearing livers. Mild cirrhosis was usually present, associated with minimal bile duct proliferation and hepatic cell nodular regeneration.

A few other tumours were observed. At 99 weeks a rat bearing a small hepatoma had in addition a spindle cell malignant tumour at the side of the head. This was diagnosed as a spindle cell sarcoma, but the possibility of spindle cell anaplasia in an acoustic duct carcinoma could not be excluded. At 113 weeks, one rat bearing large multiple hepatomas had a well differentiated adenocarcinoma of one kidney and also an early transitional cell carcinoma of the bladder. Twelve control rats receiving a diet of rat cake and malt were tumour free at death between 90 and 129 weeks (Table 1).

*Experiment* 2.—Twenty of 24 male rats survived 21 weeks of subcutaneous injection of auramine. The total dose given was 110–120 mg. (Table II). Eleven rats died between 40 and 89 weeks, 9 with subcutaneous fibrosarcomas and without

TABLE II.—*Incidence of Tumours in Wilmslow Wistar Rats Receiving Auramine by Subcutaneous Injection*

| Number of rats at start | Sex | Weeks of adminis-tration | Estimated total dose | | Tumours in rats dying at stated weeks: 40–89 | 100–113 | Percentage of tumours |
|---|---|---|---|---|---|---|---|
| 24 | M | 21 | 110–120 mg. | —Fibrosarcomas | 9/11 | 2/9 | 55 |
| | | | | —Hepatomas | 1/11 | 2/9 | 15 |

hepatomas, and one with a hepatoma but not bearing a fibrosarcoma. After 100 weeks 2 rats bore fibrosarcomas and 2 hepatomas. In this experiment three intestinal carcinomas were observed, two in the caecum at 61 and 113 weeks respectively, and one in the duodenum at 113 weeks. One of the former metastasised widely.

*Experiments* 3 *and* 4.—The maximum dose which mice would tolerate was 0·2 per cent in the diet. Hepatomas were induced in mice, but in lower incidence than in rats (Table III). In stock mice, where no hepatomas were present in the controls living to approximately the same age, hepatomas occurred in both males and females in the period 50–89 weeks. The difference between the sexes in these small numbers is not significant. The tumours were noticed with the naked eye but only attained a large size in one male mouse. No cholangiomas were seen and the degree of cirrhosis was minimal. In many of the livers there were leukaemic infiltrations.

In CBA mice, although a few hepatomas occurred spontaneously after 80 weeks, an incidence of over 50 per cent was obtained in treated mice of both sexes, the tumours appearing from 60 weeks onwards. The presence of these tumours was associated with mild bile duct proliferation and mild cellular or fibrous cirrhosis and occasionally with the formation of cholangiomatous areas, i.e. focal non-neoplastic collections of dilated bile ducts. In untreated CBA mice there was no cirrhosis. No cholangiomatous areas were seen and only in one male mouse at 114 weeks of age did mild bile duct proliferation occur.

A few other tumours occurred in stock mice but none in CBA mice In a male treated for 67 weeks there was a reticulum cell sarcoma, especially prominent in the liver and around the salivary glands, and a deeply invading adenocarcinoma of the mid-colon. In another male treated for 56 weeks there was a spindle cell sarcoma of the subcutaneous tissues of the flank. This was unlike the reticulum cell sarcoma in structure, was localised and not associated with parasitic or other visible infection. In another male treated for 79 weeks there was a benign interstitial cell tumour of the testis.

*Morphology of the tumours*

In all groups the hepatomas ranged in size from microscopical to large masses occupying the whole liver lobe. In structure they ranged from well differentiated benign tumours to anaplastic malignant-looking ones. No metastases were seen.

### DISCUSSION

Case and Pearson (1954) showed that workers engaged in the manufacture of auramine ran a risk of developing papilloma and carcinoma of the bladder. The present experiments demonstrate that this compound is carcinogenic to the liver of rats and mice when administered orally in large doses. When administered subcutaneously to rats in a small dose a few hepatomas occurred together with subcutaneous fibrosarcomas. The latter are not regarded as necessarily due to the chemical. Only one bladder tumour was found in an experimental animal—a rat—but this species divergence in location of tumours between man and rodents is well known in aromatic amine carcinogenesis.

In stock mice the hepatomas occurred in livers in which cirrhosis and bile duct proliferation was minimal. In CBA mice and rats there was mild cirrhosis and bile duct proliferation with the formation of cholangiomas. These changes were far

TABLE III.—*Induction of Hepatomas in Stock and CBA Mice Treated with Auramine*

| Number of mice at start | Sex | Mode of administration of auramine | Duration of treatment (weeks) | Approximate total quantity of auramine per mouse* | Hepatomas in mice dying at stated weeks | | | | Tumours | |
|---|---|---|---|---|---|---|---|---|---|---|
| | | | | | 50–69 | 70–89 | 90–109 | 110–119 | Total | Per cent† |
| 30 stock | 15 M. | 0·1% in diet | 52 | 1820 mg. | 1/2 | 3/5 | — | — | 4/7 | 57 |
| | 15 F. | 0·1% in diet | 52 | 1820 mg. | 2/4 | 1/6 | — | — | 3/10 | 30 |
| 16 stock | 8 M. | None‡ | — | — | 0/1 | 0/6 | 0/1 | — | 0/8 | — |
| | 8 F. | None | — | — | 0/4 | 0/2 | 0/2 | — | 0/8 | — |
| 27 CBA | 12 M. | 0·2% in diet | 52 | 3640 mg. | 3/4 | 4/8 | — | — | 7/12 | 58 |
| | 15 F. | 0·2% in diet | 52 | 3640 mg. | 1/2 | 9/12 | 1/1 | — | 11/15 | 73 |
| 90 CBA | 35 M. | None | — | — | 0/2 | 1/12 | 2/9 | 1/12 | 4/35 | 11 |
| | 55 F. | None | — | — | 0/2 | 1/18 | 2/21 | 0/14 | 3/55 | 5 |

* Assuming that each mouse eats 5 g. per day.

† In survivors to tumour age.

‡ Each mouse received weekly 0·2 c.c. arachis oil by subcutaneous injection for 52 weeks.

less advanced than in mice and rats treated with butter yellow (Orr, 1940) or in mice treated with 3,4:5,6-dibenzcarbazole (Armstrong and Bonser, 1950).

The occasional tumours occurring in other organs of excretion, i.e. the kidney, bladder and intestine were regarded as chemically induced, but much larger numbers of experimental animals would be needed to establish this with certainty.

### SUMMARY

Auramine is carcinogenic to the rat and mouse liver following oral administration. Hepatomas and fibrosarcomas occurred in the rat following subcutaneous injection. These observations lend support to the findings of Case and Pearson (1954) who reported that the manufacture of auramine was associated with an occupational hazard of tumour of the urinary bladder.

### REFERENCES

ARMSTRONG, E. C. AND BONSER, G. M.—(1950) *Brit. J. Cancer*, **4**, 203.
CASE, R. A. M. AND PEARSON, J. T.—(1954) *Brit. J. industr. Med.*, **11**, 213.
ORR, J. W.—(1940) *J. Path. Bact.*, **50**, 393.